# Long non‐coding RNA *AC018926* *.2* regulates palmitic acid exposure‐compromised osteogenic potential of periodontal ligament stem cells via the ITGA2/FAK/AKT pathway

**DOI:** 10.1111/cpr.13411

**Published:** 2023-01-31

**Authors:** Hong‐Lei Qu, Li‐Juan Sun, Xuan Li, Fen Liu, Hai‐Hua Sun, Xiao‐Tao He, Dian Gan, Yuan Yin, Bei‐Min Tian, Fa‐Ming Chen, Rui‐Xin Wu

**Affiliations:** ^1^ Department of Periodontology, State Key Laboratory of Military Stomatology and National Clinical Research Center for Oral Diseases, School of Stomatology Fourth Military Medical University Xi'an People's Republic of China; ^2^ Department of Pediatric Dentistry, College of Stomatology Xi'an Jiaotong University Xi'an People's Republic of China; ^3^ Department of General Dentistry and Emergency, State Key Laboratory of Military Stomatology and National Clinical Research Center for Oral Diseases, School of Stomatology Fourth Military Medical University Xi'an People's Republic of China

## Abstract

Although obesity has been proposed as a risk factor for periodontitis, the influence of excessive fat accumulation on the development of periodontitis and periodontal recovery from disease remains largely unknown. This study investigated the cellular response of periodontal ligament stem cells (PDLSCs) to elevated levels of a specific fatty acid, namely, palmitic acid (PA). The mechanism by which PA exposure compromises the osteogenic potential of cells was also explored. It was found that exposure of PDLSCs to abundant PA led to decreased cell osteogenic differentiation. Given that long non‐coding RNAs (lncRNAs) play a key role in the stem cell response to adverse environmental stimuli, we screened the lncRNAs that were differentially expressed in PDLSCs following PA exposure using lncRNA microarray analysis, and *AC018926.2* was identified as the lncRNA that was most sensitive to PA. Next, gain/loss‐of‐function studies illustrated that *AC018926.2* was an important regulator in PA‐mediated osteogenic differentiation of PDLSCs. Mechanistically, *AC018926.2* upregulated integrin α2 (*ITGA2*) expression and therefore activated ITGA2/FAK/AKT signalling. Further functional studies revealed that inactivation of ITGA2/FAK/AKT signalling by silencing ITGA2 counteracted the pro‐osteogenic effect induced by *AC018926.2* overexpression. Moreover, the results of bioinformatics analysis and RNA immunoprecipitation assay suggested that *AC018926.2* might transcriptionally regulate *ITGA2* expression by binding to PARP1 protein. Our data suggest that *AC018926.2* may serve as a therapeutic target for the management of periodontitis in obese patients.

## Graphical abstract

lncRNA *AC018926.2* was significantly downregulated under a palmitic acid (PA) condition, and its decreased expression was associated with PA‐compromised osteogenic differentiation of periodontal ligament stem cells (PDLSCs). Mechanistically, *AC018926.2*, mainly distributed in the nucleus, promoted the osteogenic differentiation of PDLSCs by upregulating the expression of *ITGA2* at the transcriptional level and activating the ITGA2/FAK/PI3K/AKT signalling pathway. Furthermore, *AC018926.2* might regulate the expression of *ITGA2* by interacting with PARP1 protein.

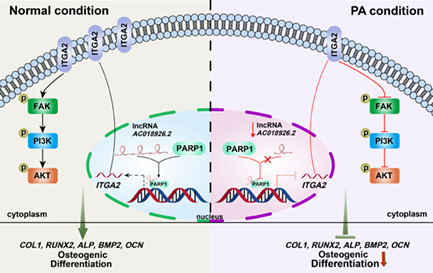

## 1 INTRODUCTION

Obesity is a serious social problem worldwide and has been widely studied because of its high prevalence and serious complications. According to the World Health Organization, 1.9 billion people were considered overweight in 2017, of which 650 million were obese, equating to 39% of the adult population, and the prevalence continues to rise.^1^ Obesity is a significant risk factor for various diseases, such as diabetes, dyslipidaemia, cardiovascular disease, osteoarthritis and periodontitis.^2^, ^3^, ^4^, ^5^

Periodontitis, initiated by dental plaque, is a multifactorial chronic inflammatory disorder of periodontal tissues. It can cause irreversible damage to the periodontium and, if left untreated, eventually lead to tooth loosening and even tooth loss.^6^, ^7^ In 2010, severe periodontitis was listed as the sixth most prevalent chronic disease in the world.^8^ Many studies have illustrated that patients with obesity were more prone to develop periodontitis and suffered from more severe periodontal destruction compared to the individuals without obesity.^9^, ^10^, ^11^ A characteristic feature of obesity is the elevated systemic and local levels of fatty acids (FAs). Recent studies have indicated that a high‐fat diet can aggravate periodontal lesions by accelerating bone destruction and impairing bone healing.^12^, ^13^, ^14^, ^15^ Previous researches on bone marrow mesenchymal stem cells, osteoblasts and osteoclasts have shown that specific FAs can regulate bone metabolism through mechanisms such as inflammation, apoptosis, autophagy and oxidative stress,^16^ resulting in impaired bone formation and enhanced bone resorption.^17^, ^18^ Periodontal ligament stem cells (PDLSCs) are a type of undifferentiated mesenchymal cell with stem cell properties isolated from PDL tissues. These cells can differentiate into cementum, alveolar bone and PDL‐like tissue, which lays a theoretical foundation for the application of PDLSCs in the regeneration of periodontal tissue.^19^ At the same time, the differentiation of PDLSCs is a complex process involving many factors. Some special conditions, such as aging, inflammation and high glucose levels, will damage the multi‐lineage potential of PDLSCs.^20^, ^21^, ^22^ Periodontal bone regeneration is currently a major challenge in periodontal therapy. However, the effect of a high‐fat condition on the osteogenic differentiation of PDLSCs and its underlying mechanisms are still poorly studied.

Long non‐coding RNAs (lncRNAs) are a class of RNA transcripts, lacking protein‐coding potential, with more than 200 nucleotides in length.^23^, ^24^ LncRNAs are involved in a variety of pathological and physiological processes.^25^, ^26^ It is worth noting that lncRNAs can serve as both potential diagnostic/prognostic biomarkers and important therapeutic targets for bone‐related diseases.^27^, ^28^, ^29^, ^30^ In recent years, scholars have made progress in uncovering the role of lncRNAs in regulating periodontal bone homeostasis. In the inflammatory microenvironment, lncRNA *GACAT2* can regulate PDLSC cementoblastic differentiation by binding to PKM1/2 proteins^22^; lncRNAs *HIF1A‐AS1* and *HIF1A‐AS2* regulate osteogenic differentiation of PDLSCs by affecting the activity of hypoxia‐inducible factor (HIIF)‐1α under hypoxia^31^; lncRNAs may also mediate force‐induced PDLSC osteogenic differentiation during orthodontic tooth movement in response to mechanical stress.^32^ Based on the findings of these studies, we hypothesize that lncRNAs play a crucial role in the damaged PDLSC osteogenic potential induced by a high‐fat environment. In this study, we established a high‐fat condition with a specific FA, palmitic acid (PA), to monitor the PA‐induced changes in the osteogenic differentiation of PDLSCs. A key lncRNA, *AC018926.2*, was screened out and identified to be downregulated in PDLSCs exposed to PA compared to those under a normal condition, and it was shown to be involved in regulating PDLSC osteogenic potential in an environment with PA. Mechanistically, the results revealed that *AC018926.2* elicited its biological functions by increasing the expression level of integrin α2 (*ITGA2*) and therefore activating ITGA2/FAK/AKT signalling. Overall, our research suggested that *AC018926.2* may serve as a novel therapeutic target for the recovery of periodontitis in obese individuals.

## 2 MATERIALS AND METHODS

Detailed materials and methods are described in the Supplementary Information.

## 3 RESULTS

### 3.1 PA exposure compromised the osteogenic differentiation of PDLSCs

Primary human PDLSCs were successfully isolated from the PDL tissues of 6 fresh teeth (Figure S1A). The results of the colony‐forming unit (CFU) (Figure S1B) and CCK‐8 (Figure S1C) assays confirmed that the PDLSCs have the ability to proliferate and multiply. The cells could also differentiate into osteogenic, adipogenic and chondrogenic lineages, as verified by Alizarin red S staining, Oil red O staining and Alcian blue staining, respectively (Figure S1D). Flow cytometry analysis (Figure S1E) showed that these PDLSCs positively expressed CD105, CD44, CD90 and CD146 and negatively expressed CD45 and CD34. Then, these cells were used for the following in vitro experiments.

**FIGURE 1:**
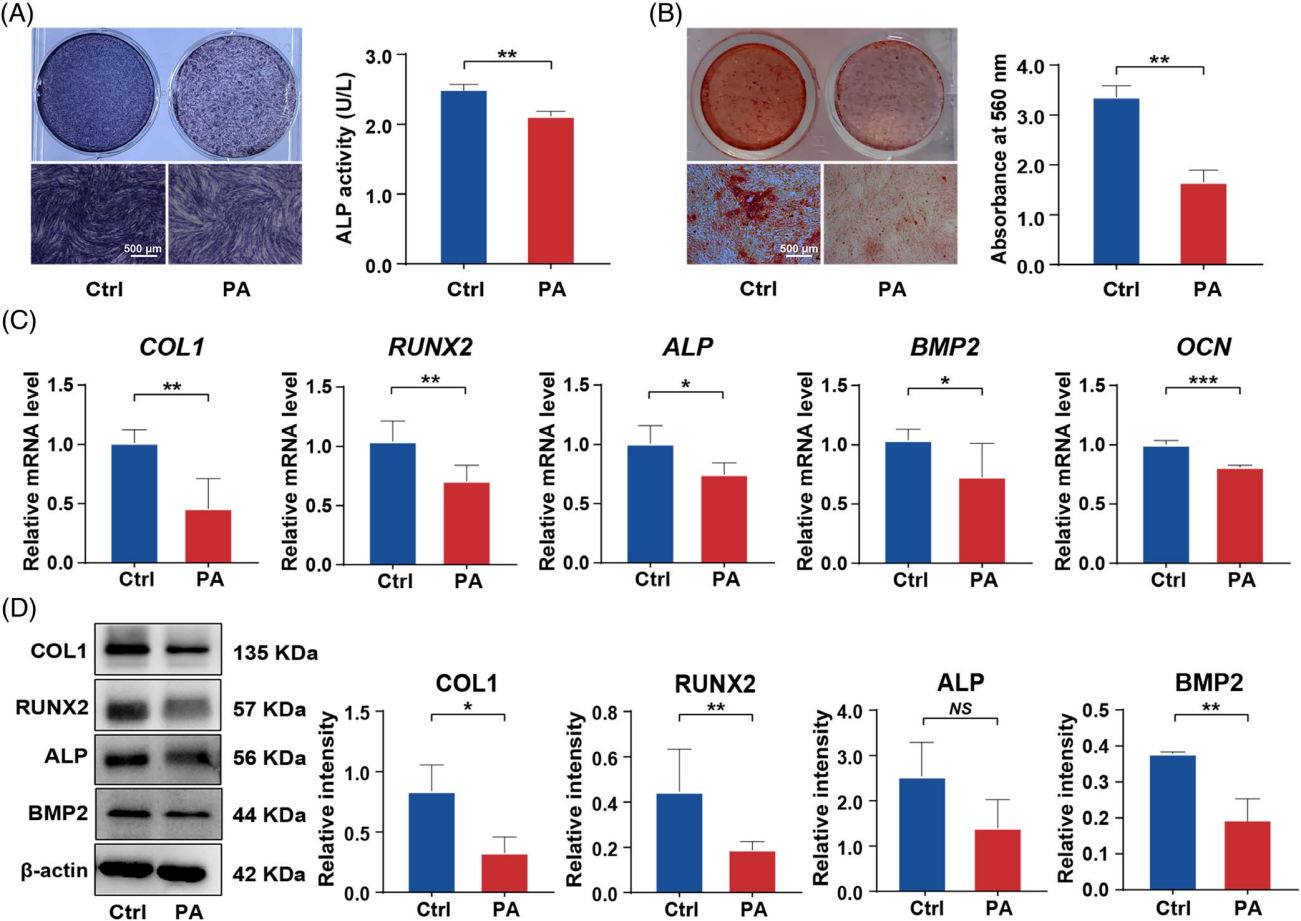
The osteogenic differentiation of PDLSCs was suppressed when they were exposed to PA for at least 14 days. The cells were incubated in osteogenic medium with BSA (Ctrl) or osteogenic medium with PA. (A) Representative images of ALP staining and quantification of ALP activity of PDLSCs at Day 14 of culturing in Ctrl or PA conditions (scale bar: 500 μm). (B) Representative images of Alizarin red S staining and quantitative analysis of the calcium mineral deposits formed by PDLSCs at Day 21 of culturing in Ctrl or PA conditions (scale bar: 500 μm). (C) Expression levels of the osteogenesis‐related genes *COL1*, *RUNX2*, *ALP*, *BMP2* and *OCN* in PDLSCs after 14 days of exposure to PA measured by qRT‐PCR. (D) Expression levels of the osteogenesis‐related proteins COL1, RUNX2, ALP and BMP2 in PDLSCs after 14 days of exposure to PA determined by Western blot analysis. All experiments were performed with 3 biological replicates. Data are presented as the mean ± SD (*n* = 3). *p < 0.05, **p < 0.01, and ***p < 0.001 represent significant differences between the indicated columns, while *NS* represents no significant difference.

In order to build a high‐fat condition, PA was added to the osteogenic medium (PA group) to adjust its final concentration to 200 μM, and the control group was treated with the same amount of solvent control (FA‐free bovine serum albumin, BSA). The use of PA at a concentration of 200 μM was based on previously published literature.^33^, ^34^, ^35^, ^36^ Then, we systematically monitored the effects of PA exposure on the osteogenic potential of PDLSCs through a series of related experiments. We found that ALP activity was significantly decreased in the PA group compared with the control group, as indicated by ALP staining and quantification (Figure 1A). Alizarin red S staining and quantification on Day 21 showed that extracellular mineralization was also reduced in the PA group (Figure 1B). Meanwhile, when the cells were exposed to PA for 14 days, the expression levels of osteogenesis‐related genes *COL1*, *RUNX2*, *ALP*, *BMP2* and *OCN* were dramatically impaired, as demonstrated by a quantitative real‐time polymerase chain reaction (qRT‐PCR) assay (Figure 1C). Moreover, Western blot analysis revealed that the levels of osteogenesis‐related proteins COL1, RUNX2 and BMP2 were significantly decreased in the PA group on Day 14 (Figure 1D). These results indicate that PA exposure could impair the osteogenic differentiation of PDLSCs.

### 3.2 lncRNA microarray analysis and qRT‐PCR validation

**FIGURE 2:**
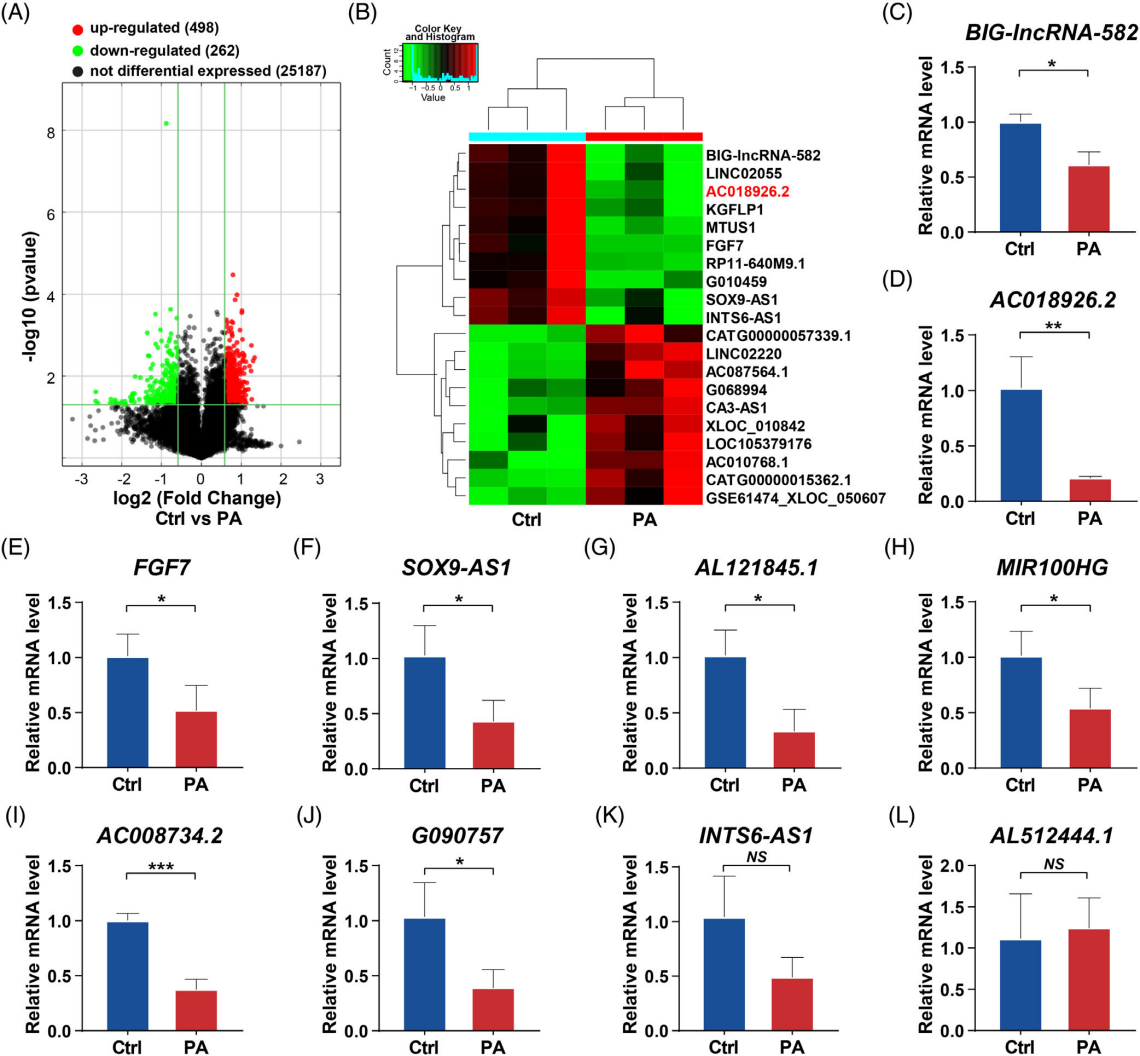
The expression profile of lncRNAs in PDLSCs incubated under control or PA conditions and validation of the microarray results. The samples were cultured in osteogenic medium with BSA (Ctrl) or osteogenic medium with PA for 14 days. (A) Volcano plots of the differentially expressed lncRNAs (fold change >1.5 and adjusted *p* < 0.05) in 3 pairs of PDLSCs when comparing the PA and Ctrl groups. (B) Heatmap of the top 10 downregulated and top 10 upregulated lncRNAs in the PDLSCs of the PA group compared with those of the Ctrl group. Red: upregulated lncRNAs in the PA group; green: downregulated lncRNAs in the PA group. (C–L) Verification of the 10 selected lncRNAs, that is, *BIG‐lncRNA‐582* (C), *AC018926.2* (D), *FGF7* (E), *SOX9‐AS1* (F), *AL121845.1* (G), *MIR100HG* (H), *AC008734.2* (I), *G090757* (J), *INTS6‐AS1* (K) and *AL512444.1* (L), by qRT‐PCR assays. All experiments were performed with 3 biological replicates. Data are presented as the mean ± SD (*n* = 3). *p < 0.05, **p < 0.01 and ***p < 0.001 represent significant differences between the indicated columns, while *NS* represents no significant difference.

As the osteogenic differentiation of PDLSCs was significantly inhibited after 14 days of PA exposure, we performed a microarray analysis to identify the expression profiles of lncRNAs in PDLSCs 14 days after osteogenic induction when cells were exposed to PA (PA group) or BSA (Ctrl group). A total of 760 lncRNAs (*p* < 0.05 and fold change >1.5) were differentially expressed between the two groups, of which 262 were significantly downregulated and 498 were significantly upregulated in cells in the PA group compared with those in the Ctrl group (Figure 2A). The heatmap exhibited the symbols of the top 10 downregulated lncRNAs and top 10 upregulated lncRNAs in the PA group (Figure 2B). According to the absolute value of the fold change, 10 lncRNAs, *BIG‐lncRNA‐582*, *AC018926.2*, *FGF7*, *SOX9‐AS1*, *INTS6‐AS1*, *MIR100HG*, *AC008734.2*, *G090757*, *AL121845.1* and *AL512444.1*, met the screening conditions and were selected to verify the microarray results. The screening criteria were as follows: RNA length < 2000 nt, a raw intensity >100, and inclusion in a database (in GENCODE or RefSeq public databases). Based on the qRT‐PCR assay, eight of the 10 lncRNAs (*BIG‐lncRNA‐582*, *AC018926.2*, *FGF7*, *SOX9‐AS1*, *MIR100HG*, *AC008734.2*, *G090757* and *AL121845.1*) showed the same trend as the microarray results, that is, significantly lower expression in the PA group than in the Ctrl group (Figure 2C–J), while the other 2 lncRNAs (*INTS6‐AS1* and *AL512444.1*) showed no difference between the two groups (Figure 2K, L). According to the verification results, *AC018926.2*, which overlapped with no coding transcripts, was the most downregulated lncRNA in the PA group compared to the Ctrl group (fold change = 4.9) (highlighted in red on the heatmap in Figure 2B); thus, its biological functions and underlying molecular mechanisms in the process of osteogenic differentiation of PDLSCs were explored in this study.

### 3.3 Forced expression of *AC018926.2* reversed PA‐compromised osteogenic differentiation of PDLSCs

**FIGURE 3:**
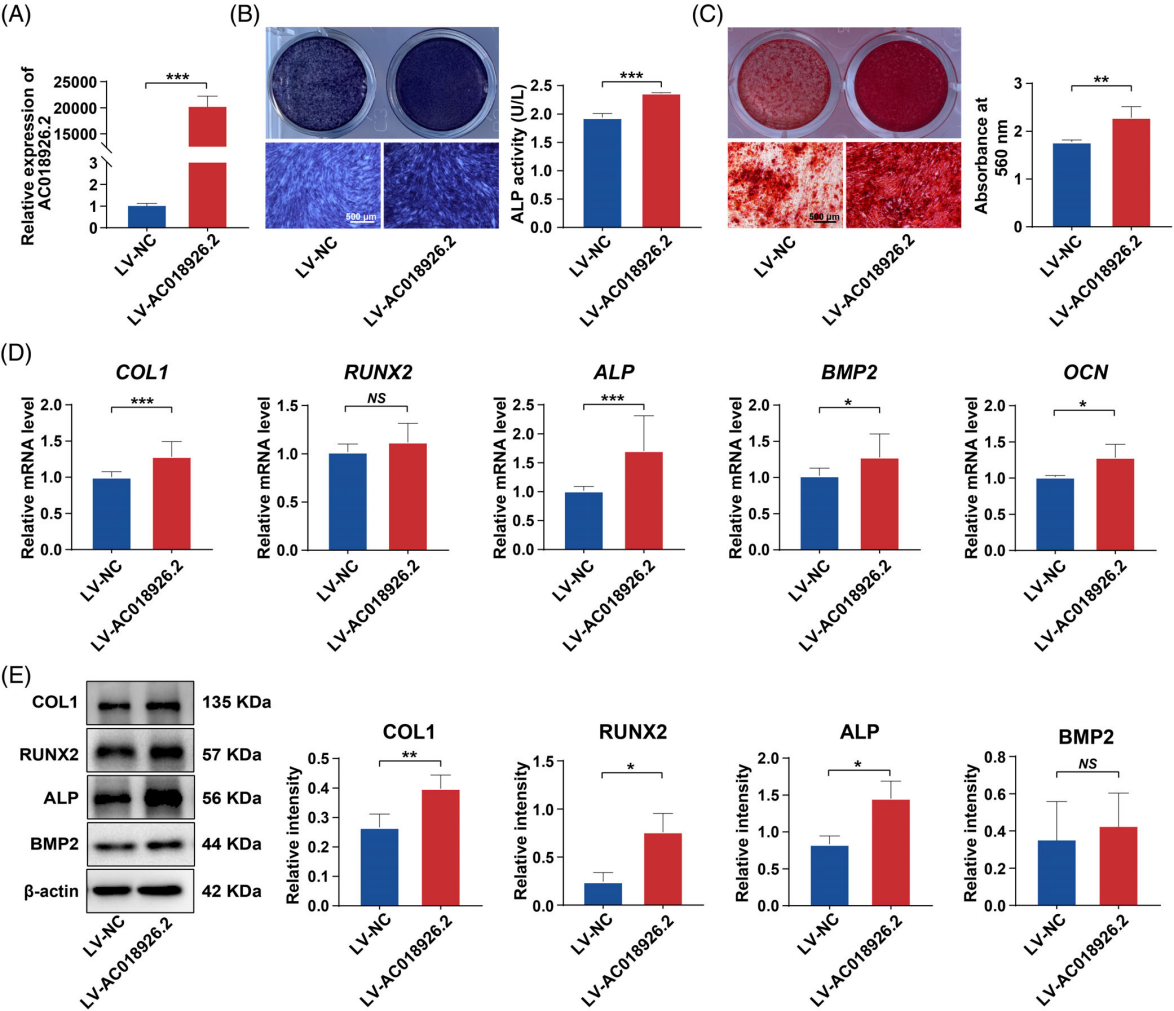
Upregulation of *AC018926.2* rescued the PA‐induced osteogenic damage to PDLSCs. The cells were incubated in osteogenic medium with PA, and *AC018926.2* was overexpressed in PDLSCs with lentivirus. (A) Overexpression efficiency of *AC018926.2* in PDLSCs measured by qRT‐PCR. (B) Representative images of ALP staining and quantification of ALP activity in PDLSCs transfected with LV‐NC and LV‐*AC018926.2* after osteogenic induction for 14 days (scale bar: 500 μm). (C) Representative images of Alizarin red S staining and quantitative analysis of the calcium mineral deposits formed by PDLSCs transfected with LV‐NC and LV‐*AC018926.2* after osteogenic induction for 21 days (scale bar: 500 μm). (D) The effect of *AC018926.2* overexpression on the expression levels of the osteogenesis‐related genes *COL1*, *RUNX2*, *ALP*, *BMP2* and *OCN* in PDLSCs following 14 days of osteogenic induction measured by qRT‐PCR. (E) The effect of *AC018926.2* overexpression on the osteogenesis‐related proteins COL1, RUNX2, ALP and BMP2 in PDLSCs following 14 days of osteogenic induction determined by Western blot analysis. All experiments were performed with 3 biological replicates. Data are presented as the mean ± SD (*n* = 3). *p < 0.05, **p < 0.01 and ***p < 0.001 represent significant differences between the indicated columns, while *NS* represents no significant difference.

To investigate the role of *AC018926.2* in PDLSC osteogenic differentiation, we first transfected lentivirus containing full‐length *AC018926.2* into PDLSCs to overexpress *AC018926.2* under a PA condition because this lncRNA was downregulated after PA exposure. qRT‐PCR showed a nearly 20,000‐fold increase in the expression level of *AC018926.2*, indicating successful overexpression (Figure 3A). ALP staining and quantitative analysis demonstrated that ALP production significantly increased in the *AC018926.2*‐overexpressing group (LV‐*AC018926.2* group) compared with the control group (LV‐NC group) (Figure 3B). Similarly, Alizarin red S staining and quantification indicated that a higher level of calcium deposition was observed in the LV‐*AC018926.2* group (Figure 3C). These results were further confirmed by the quantitative evaluation of osteogenesis‐related genes and proteins. As shown in Figure 3D, the gene expression of *COL1*, *ALP*, *BMP2* and *OCN* was markedly enhanced when *AC018926.2* was upregulated. Western blot experiments also showed higher levels of the COL1, RUNX2 and ALP proteins in the LV‐*AC018926.2* group (Figure 3E). The various analyses of osteogenic function revealed that the overexpression of *AC018926.2* significantly alleviated PA‐induced osteogenic impairment of PDLSCs.

### 3.4 Reduced expression of *AC018926.2* impaired the osteogenic differentiation of PDLSCs under a normal condition

**FIGURE 4:**
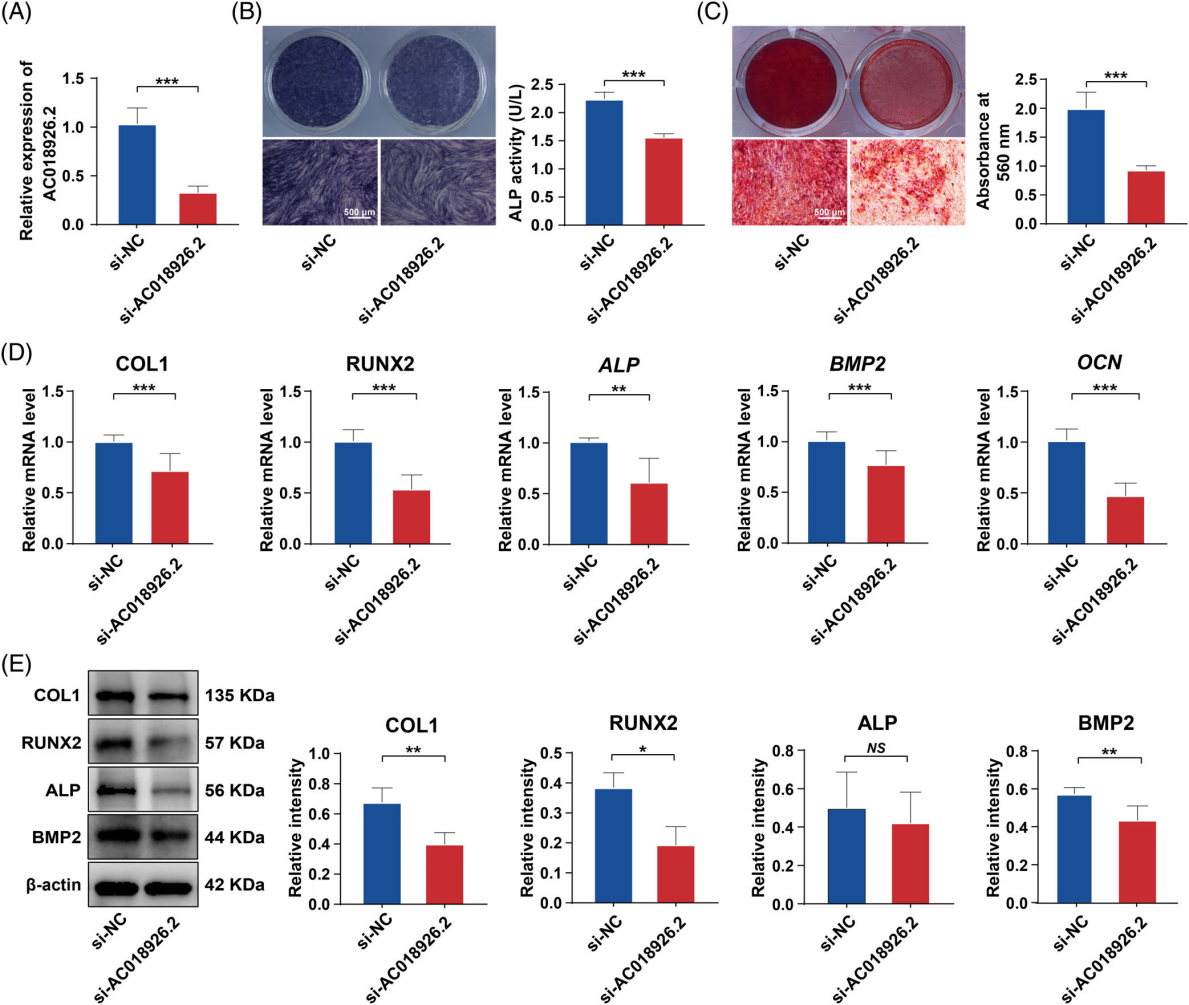
Downregulation of *AC018926.2* inhibited PDLSC osteogenic differentiation under a normal condition. The cells were incubated in normal osteogenic medium, and *AC018926.2* was knocked down with specific siRNA. (A) Knockdown efficiency of *AC018926.2* in PDLSCs measured by qRT‐PCR. (B) Representative images of ALP staining and quantification of ALP activity in PDLSCs transfected with si‐NC and si‐*AC018926.2* after osteogenic induction for 14 days (scale bar: 500 μm). (C) Representative images of Alizarin red S staining and quantitative analysis of the calcium mineral deposits formed by PDLSCs transfected with si‐NC and si‐*AC018926.2* after osteogenic induction for 21 days (scale bar: 500 μm). (D) The effect of *AC018926.2* knockdown on the expression levels of the osteogenesis‐related genes *COL1*, *RUNX2*, *ALP*, *BMP2* and *OCN* in PDLSCs following 14 days of osteogenic induction measured by qRT‐PCR. (E) The effect of *AC018926.2* knockdown on the osteogenesis‐related proteins COL1, RUNX2, ALP and BMP2 in PDLSCs following 14 days of osteogenic induction determined by Western blot analysis. All experiments were performed with 3 biological replicates. Data are presented as the mean ± SD (*n* = 3). *p < 0.05, **p < 0.01 and ***p < 0.001 represent significant differences between the indicated columns, while *NS* represents no significant difference.

In addition to gain‐of‐function assays, we also performed loss‐of‐function assays. siRNA transfection technology was utilized to knock down the expression of *AC018926.2* in PDLSCs under a normal condition, and Figure 4A shows the knockdown efficiency. Then, we examined the effect of silencing *AC018926.2* on the osteogenic potential of PDLSCs. Consistent with the overexpression experiments, the results demonstrated that reduced *AC018926.2* expression significantly suppressed PDLSC osteogenic differentiation. ALP staining and quantification revealed that ALP activity was markedly decreased in the group in which *AC018926.2* was silenced (si‐*AC018926.2* group) compared with the control group (si‐NC group) (Figure 4B). Alizarin red S staining and quantitative analysis showed a similar trend to ALP activity (Figure 4C). In addition, the expression levels of all the tested genes, including *COL1*, *RUNX2*, *ALP*, *BMP2* and *OCN*, were significantly reduced upon *AC018926.2* knockdown (Figure 4D). Meanwhile, the protein expression levels of COL1, RUNX2 and BMP2 were also affected by *AC018926.2* downregulation, that is, the levels were decreased with the reduction in *AC018926.2* (Figure 4E). The above gain/loss‐of‐function assays suggest that *AC018926.2* plays an important role in the impairment of PDLSC osteogenic differentiation induced by PA exposure.

### 3.5 *AC018926.2* was positively correlated with the activity of the ITGA2/FAK/PI3K/AKT pathway

**FIGURE 5:**
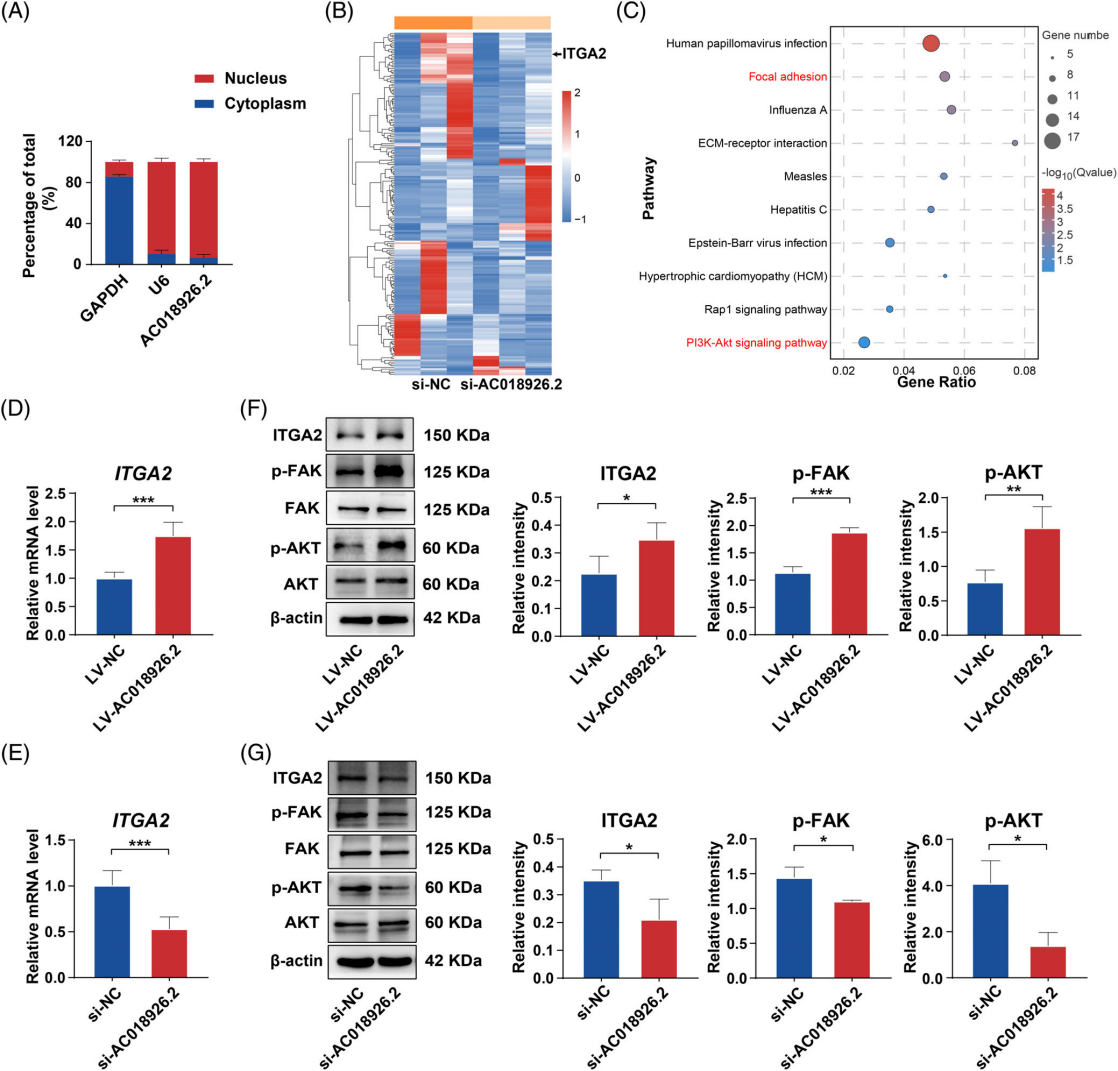
*AC018926.2* overexpression/knockdown affected the activity of the ITGA2/FAK/PI3K/AKT pathway by regulating *ITGA2* expression. (A) Percentages of nuclear and cytoplasmic *AC018926.2* in PDLSCs measured by qRT‐PCR (*U6* and *GAPDH* served as the nuclear and cytoplasmic controls, respectively). (B, C) Transcriptomic analysis of PDLSCs comparing the *AC018926.2* knockdown group and the control group. The samples were transfected with si‐NC and si‐*AC018926.2* and cultured in osteogenic medium for 14 days for RNA sequencing. (B) Heatmap of coding genes with significantly altered expression (fold change >2 and adjusted *p* < 0.05) following *AC018926.2* knockdown in 3 pairs of PDLSCs. Red: Upregulated genes in the *AC018926.2* knockdown PDLSCs; blue: Downregulated genes in the *AC018926.2* knockdown PDLSCs. (C) The top 10 enriched pathways significantly associated with *AC018926.2* knockdown illustrated by KEGG analysis. The FAK/PI3K/AKT signalling pathway was notably affected. (D–G) *AC018926.2* affects the activity of the ITGA2/FAK/AKT pathway. (D, E) The effect of *AC018926.2* upregulation/downregulation on the expression of *ITGA2* at the RNA level measured by qRT‐PCR. (F) *AC018926.2* overexpression activated ITGA2/FAK/AKT signalling. The protein expression levels of ITGA2, p‐FAK, total FAK, p‐AKT and total AKT in PDLSCs with (LV‐ *AC018926.2*) or without (LV‐NC) *AC018926.2* overexpression were assessed with Western blot analysis. (G) *AC018926.2* knockdown inhibited ITGA2/FAK/AKT signalling. The protein expression levels of ITGA2, p‐FAK, total FAK, p‐AKT and total AKT in PDLSCs with (si‐*AC018926.2*) or without (si‐NC) *AC018926.2* knockdown were assessed with Western blot analysis. The relative intensity of ITGA2 was normalized to β‐actin, and the relative intensity of p‐FAK and p‐AKT was normalized to respective total FAK and total AKT (F, G). All experiments were performed with 3 biological replicates. Data are presented as the mean ± SD (*n* = 3). *p < 0.05, **p < 0.01 and ***p < 0.001 represent significant differences between the indicated columns.

Because the regulatory mechanisms of lncRNAs are associated with their subcellular localization,^37^ we conducted subcellular fractionation assay to evaluate the distribution of *AC018926.2*. We found that *AC018926.2* was primarily observed in the nuclear fraction and was less prevalent in the cytoplasm (Figure 5A). This suggested that *AC018926.2* may regulate the transcription of target genes.^38^

To better understand how *AC018926.2* affects PDLSC osteogenic differentiation, we performed RNA transcriptome sequencing following *AC018926.2* knockdown in PDLSCs. As shown in the heatmap, 196 differentially expressed genes were identified (fold change >2 and *p* < 0.05) in the PDLSCs from the si‐*AC018926.2* group compared to those from the si‐NC group, including 138 downregulated genes and 58 upregulated genes (Figure 5B). Furthermore, the Kyoto Encyclopedia of Genes and Genomes (KEGG) pathway analysis was utilized to investigate the signalling pathways that may be impacted by *AC018926.2*. Figure 5C shows the top 10 enriched signalling pathways, among which the focal adhesion and phosphatidylinositol 3‐kinase (PI3K)/AKT signalling pathways, which are closely related to cell osteogenesis, were significantly downregulated in the si‐*AC018926.2* group and identified.^39^, ^40^ Therefore, we hypothesized that silence of *AC018926.2* could inhibit the osteogenic process of PDLSCs by inactivating the FAK/PI3K/AKT signalling pathway. In this pathway, integrins induce the phosphorylation of focal adhesion kinase (FAK), and then, p‐FAK activates PI3K/AKT.^39^ ITGA2 is a member of the integrin family, and the KEGG analysis and previous studies demonstrated that ITGA2 activates the downstream proteins of FAK signalling and then regulates the osteogenic differentiation of stem cells.^41^, ^42^, ^43^, ^44^ Consistent with the sequencing results, *AC018926.2* could regulate *ITGA2* expression at the transcriptional level (Figure 5D, E). Then, we used Western blot analysis to detect the protein levels of ITGA2, p‐FAK, total FAK, p‐AKT and total AKT in PDLSCs after overexpression and knockdown of *AC018926.2*. The protein expression of ITGA2, p‐FAK and p‐AKT increased when *AC018926.2* was overexpressed but decreased when *AC018926.2* was knocked down (Figure 5F, G). These results suggested that *AC018926.2* positively affects the activity of the ITGA2/FAK/PI3K/AKT pathway.

Meanwhile, Western blot analysis was conducted to assess the protein levels of ITGA2, p‐FAK, total FAK, p‐AKT and total AKT in PDLSCs after PA exposure. It was found that the protein expression of ITGA2, p‐FAK and p‐AKT was significantly reduced, indicating that PA exposure inhibited the activity of the ITGA2/FAK/PI3K/AKT pathway (Figure S2A, B).

### 3.6 ITGA2 knockdown attenuated the *AC018926.2* overexpression‐mediated enhancement of PDLSC osteogenic differentiation

**FIGURE 6:**
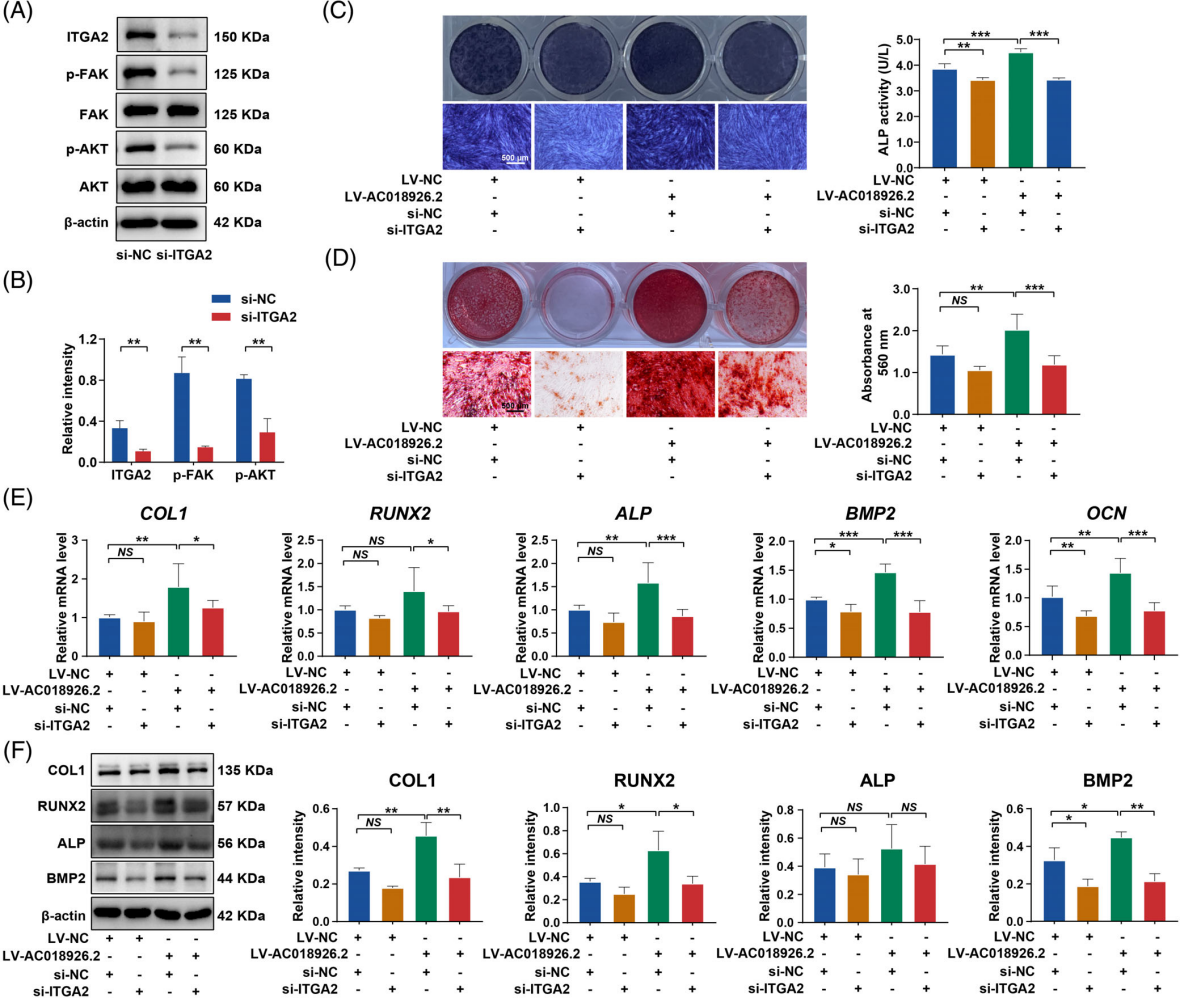
ITGA2 inhibition impaired the *AC018926.2* overexpression‐rescued osteogenic differentiation of PDLSCs under the PA condition. (A, B) ITGA2, p‐FAK, total FAK, p‐AKT and total AKT protein levels in the control and si‐ITGA2 groups measured by Western blot analysis. The relative intensity of ITGA2 was normalized to β‐actin, and the relative intensity of p‐FAK and p‐AKT was normalized to respective total FAK and total AKT. The cells were incubated in normal medium transfected with si‐ITGA2. (C) Representative images of ALP staining and quantification of ALP activity in PDLSCs after osteogenic induction for 14 days (scale bar: 500 μm). (D) Representative images of Alizarin red S staining and quantitative analysis of the calcium mineral deposits formed by PDLSCs after osteogenic induction for 21 days (scale bar: 500 μm). (E) The effect of ITGA2 silence on the expression levels of the osteogenesis‐related genes *COL1*, *RUNX2*, *ALP*, *BMP2* and *OCN* in *AC018926.2* overexpressed PDLSCs following 14 days of osteogenic induction measured by qRT‐PCR. (F) The effect of ITGA2 silence on the osteogenesis‐related proteins COL1, RUNX2, ALP and BMP2 in *AC018926.2* overexpressed PDLSCs following 14 days of osteogenic induction determined by Western blot analysis. The cells were incubated in osteogenic medium with PA and co‐transfected with LV‐*AC018926.2* and si‐ITGA2 (C–F). All experiments were performed with 3 biological replicates. Data are presented as the mean ± SD (*n* = 3). *p < 0.05, **p < 0.01 and ***p < 0.001 represent significant differences between the indicated columns, while *NS* represents no significant difference.

Various rescue experiments were conducted to verify whether *AC018926.2* affects the osteogenic potential of PDLSCs by regulating the ITGA2/FAK/PI3K/AKT pathway. We treated *AC018926.2*‐overexpressing PDLSCs with si‐ITGA2 to inactivate the ITGA2/FAK/PI3K/AKT pathway and evaluated the osteogenic differentiation potential of PDLSCs under a PA condition. The levels of the ITGA2, p‐FAK and p‐AKT proteins were significantly decreased following ITGA2 knockdown (Figure 6A, B). As shown by ALP staining and quantification, inhibition of the ITGA2/FAK/PI3K/AKT pathway impaired the enhancement of ALP activity caused by *AC018926.2* overexpression (Figure 6C). Similarly, administration of si‐ITGA2 led to a decrease in the secreted mineralization of *AC018926.2*‐overexpressing PDLSCs (Figure 6D). Consistently, qRT‐PCR analysis demonstrated that ITGA2 knockdown attenuated the elevated effects of *AC018926.2* overexpression on the gene levels of *COL1*, *RUNX2*, *ALP*, *BMP2* and *OCN* (Figure 6E). The Western blot results also revealed that the enhanced protein levels of COL1, RUNX2 and BMP2 induced by *AC018926.2* overexpression were impaired by ITGA2/FAK/PI3K/AKT pathway inhibition (Figure 6F). These results suggested that *AC018926.2* regulates the osteogenic differentiation of PDLSCs by activating ITGA2/FAK/PI3K/AKT signalling.

To further verify that the FAK/PI3K/AKT pathway was involved in the process by which *AC018926.2* influenced PDLSC osteogenic differentiation, we also used siRNA technology to transfect *AC018926.2*‐overexpressing PDLSCs with si‐FAK and evaluated the osteogenic potential of PDLSCs. The protein levels of FAK, p‐FAK and p‐AKT were significantly decreased following FAK silencing (Figure S3A, B). As expected, the increased ALP activity (Figure S3C), mineralization nodules (Figure S3D), osteogenesis‐related genes (*RUNX2*, *ALP*, *BMP2* and *OCN*) (Figure S3E), and osteogenesis‐related proteins (COL1, RUNX2, ALP and BMP2) (Figure S3F) induced by *AC018926.2* overexpression were evidently suppressed following FAK knockdown.

### 3.7 *AC018926.2* might affect *ITGA2* expression at the transcriptional level by binding to poly (ADP‐ribose) polymerase 1 (PARP1)

**FIGURE 7:**
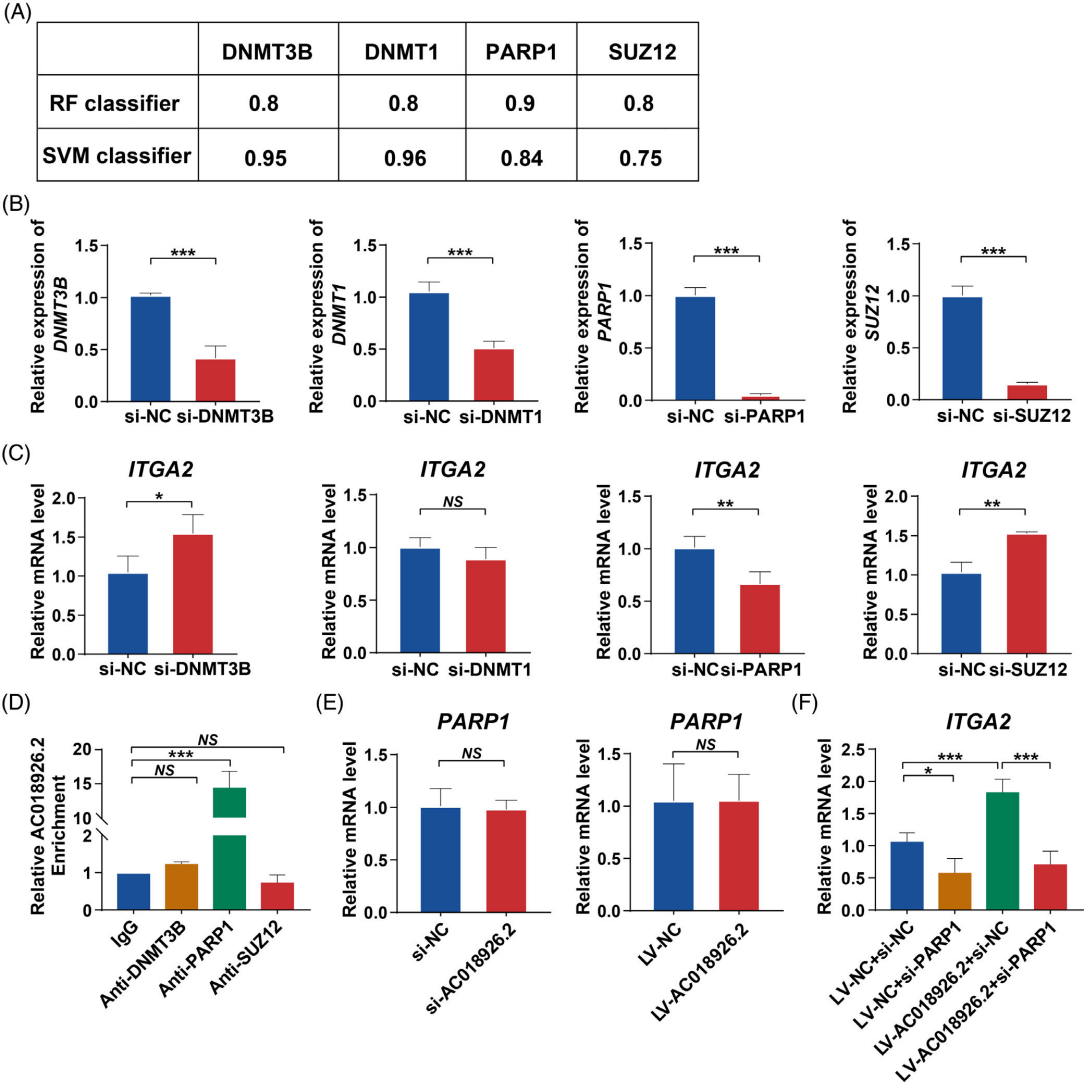
*AC018926.2*‐mediated *ITGA2* expression by interacting with the PARP1 protein. (A) The possibility of *AC018926.2* interacting with DMNT3B, DMNT1, PARP1 and SUZ12, was identified using the RPIseq database. Random forest (RF) and support vector machine (SVM) models with probabilities greater than 0.5 were considered positive. (B) The knockdown efficiency of DMNT3B, DMNT1, PARP1 and SUZ12 in PDLSCs measured by qRT‐PCR assay. (C) Effect of DMNT3B, DMNT1, PARP1 and SUZ12 knockdown on *ITGA2* expression at the RNA level. (D) The binding relationship between *AC018926.2* and the proteins (DMNT3B, PARP1 and SUZ12) in PDLSCs determined by RIP assay. IgG antibodies were used as the control. (E) The mRNA level of *PARP1* following *AC018926.2* downregulation or upregulation assessed by qRT‐PCR assay. (F) qRT‐PCR showing the changes in *ITGA2* expression after *AC018926.2* overexpression and *PARP1* knockdown. Data are presented as the mean ± SD (*n* = 3). *p < 0.05, **p < 0.01 and ***p < 0.001 represent significant differences between the indicated columns, while *NS* represents no significant difference.

As shown above, *AC018926.2* regulates the expression of *ITGA2* at the transcriptional level, and one of the ways in which lncRNAs function is by binding proteins to regulate mRNA expression.^38^, ^45^ Therefore, we hypothesized that *AC018926.2* interacts with specific proteins to achieve this result. To identify potential *AC018926.2*‐interacting proteins, we performed a sequence‐based RNA‐protein interaction prediction and identified a cohort of candidate proteins (http://service.tartaglialab.com/page/catrapid_group). Among the candidate proteins, DNA methylation transferase 3B (DNMT3B), DNA methylation transferase 1 (DNMT1), PARP1 and suppressor of zeste 12 homologue (SUZ12) have been reported to possibly regulate the expression of ITGA2.^46^, ^47^, ^48^ Then, we used another website to predict the likelihood of *AC018926.2* interacting with these four proteins (http://pridb.gdcb.iastate.edu/RPISeq/index.html). The results of the bioinformatics analysis confirmed that the four proteins had a high probability of binding to *AC018926.2*, as the random forest (RF) and support vector machine (SVM) values were higher than 0.5 (Figure 7A). Therefore, these four proteins were selected for further analysis. To determine the effects of DNMT3B, DNMT1, PARP1 and SUZ12 on *ITGA2* transcription levels, we transfected PDLSCs with DNMT3B, DNMT1, PARP1 and SUZ12 siRNAs, and the knockdown efficiency is shown in Figure 7B. qRT‐PCR assays revealed that DNMT3B and SUZ12 knockdown evidently enhanced the gene level of *ITGA2*, PARP1 knockdown notably suppressed the gene level of *ITGA2*, while DNMT1 knockdown did not influence the gene level of *ITGA2* (Figure 7C). Subsequently, DNMT1 was excluded, and RNA immunoprecipitation (RIP) assays were used to detect the interaction between the remaining three proteins, DNMT3B, SUZ12 and PARP1 and *AC018926.2*. The results showed that *AC018926.2* was significantly enriched in PARP1 antibody when compared to the control immunoglobulin G (IgG) antibody (14.5‐fold change compared to IgG by qRT‐PCR from RIP assay) (Figure 7D). Meanwhile, the gene expression level of *PARP1* was not affected by *AC018926.2* downregulation or upregulation (Figure 7E). These data indicated that *AC018926.2* specifically interacted with PARP1. To further verify that *AC018926.2* influences the expression of *ITGA2* by binding to PARP1, we overexpressed *AC018926.2* and silenced PARP1 to examine the expression of *ITGA2* measured by qRT‐PCR. We found that PARP1 downregulation impaired the impact that *AC018926.2* upregulation had on enhancing the mRNA level of *ITGA2* (Figure 7F).

## 4 DISCUSSION

In the present study, we observed that PA exposure impaired the osteogenic potential of PDLSCs. Moreover, we found that *AC018926.2* was significantly downregulated in PDLSCs exposed to PA condition, which was associated with the compromised osteogenic potential of PDLSCs. Mechanistically, as an important regulator in the osteogenesis process, *AC018926.2*, mainly distributed in the nucleus, promoted the osteogenic differentiation of PDLSCs by upregulating the expression of *ITGA2* at the transcriptional level and activating the ITGA2/FAK/PI3K/AKT signalling pathway. Furthermore, *AC018926.2* might regulate the expression of *ITGA2* by interacting with PARP1.

Increasing evidence has confirmed that obesity and obesity‐related complications are associated with the onset, progression and recovery of periodontitis.^2^, ^3^, ^49^, ^50^ However, the specific mechanism is still under investigation. In obese patients, plasma‐free FAs, derived from adipose tissues and diet, are elevated and have proinflammatory effects.^51^ Therefore, increased levels of free FAs in obese patients might contribute to the association between obesity and periodontal destruction.^52^ PA is the most abundant saturated FA in daily diet and adipose tissues,^53^ and elevated PA levels in the serum can be converted into increased PA levels in the bone tissues.^33^ Based on this, we focused on how PA will affect PDLSC osteogenic differentiation. Nevertheless, there remains controversy in the previous studies regarding the effect of PA on bone metabolism. Some studies suggest that PA exhibits high lipotoxicity and can negatively affect the osteogenic differentiation of osteoblasts,^18^, ^33^, ^54^ while other studies suggest that PA affects bone homeostasis mainly by influencing osteoclasts rather than osteoblasts.^48^, ^49^, ^50^ According to our present study supported by systematical observation, when exposed to PA, the osteogenic potential of PDLSCs was notably impaired (Figure 1). The discrepancies between previous studies may be partially related to different PA exposure duration. Considering obesity is a chronic state, to better simulate in vivo situation, we adopted the prolonged PA exposure (at least 14 days as was used in our study) rather than the 6–8 days incubation protocol in most previous studies.^34^, ^35^, ^36^

LncRNAs are important components of non‐coding RNAs. It has been found that lncRNAs can regulate gene expression at the transcriptional, posttranscriptional and epigenetic levels through various mechanisms, thereby positively or negatively influencing the osteogenic differentiation of PDLSCs under both physiological and pathological conditions.^30^, ^55^, ^56^ However, whether lncRNAs are involved in the regulation of the damaged osteogenic potential of PDLSCs under a PA condition remains to be determined. We explored the lncRNAs that are differentially expressed in PA‐exposed PDLSCs and their controls by microarray analysis as they may be potential targets for periodontal bone regeneration. To the best of our knowledge, there have been few similar microarray screenings of lncRNAs in PDLSCs under PA exposure. Nevertheless, not all up/downregulated lncRNAs under a PA condition play a role in the complex regulatory process. Therefore, the identification of potential functional lncRNAs is a challenging task. After validating the results of the microarray analysis, among the 10 verified lncRNAs, *AC018926.2* was the most reduced in the PA condition compared with the normal condition (Figure 2). This novel lncRNA attracted our interest because of its unexplored possible function in the process of osteogenesis. We then conducted gain/loss‐of‐function studies to assess the role of *AC018926.2* in regulating the osteogenic differentiation of PDLSCs. The results showed that *AC018926.2* overexpression significantly enhanced the osteogenesis, while *AC018926.2* knockdown notably impaired the osteogenesis of PDLSCs (Figures 3 and 4). Based on these observations, *AC018926.2* was identified as an active regulator in the osteogenic differentiation of PDLSCs.

From the results of RNA sequencing and KEGG analysis, we found that the FAK and PI3K/AKT pathway were significantly downregulated following *AC018926.2* knockdown (Figure 5C). Previous studies have shown that activation of the FAK/PI3K/AKT pathway is positively associated with cell osteogenic differentiation.^39^, ^40^ As an important member of the integrin family, ITGA2 has been reported to enhance the osteogenic capacity of cells by activating the FAK signalling pathway.^43^, ^44^ Furthermore, the RNA sequencing results showed that *ITGA2* expression was decreased after *AC018926.2* knockdown (Figure 5B). In addition, qRT‐PCR assays confirmed that the gene level of *ITGA2* was affected by overexpression/knockdown of *AC018926.2* (Figure 5D, E). Therefore, we speculated that *AC018926.2* promoted the osteogenic differentiation of PDLSCs by activating the ITGA2/FAK/PI3K/AKT pathway through regulating the expression of *ITGA2*. As expected, the ITGA2/FAK/PI3K/AKT pathway was activated following *AC018926.2* upregulation and inactivated following *AC018926.2* downregulation (Figure 5F, G). When the pathway was inhibited by specific siRNAs for ITGA2, the ability of overexpressed *AC018926.2* to enhance osteogenic differentiation of PDLSCs under PA exposure was blocked (Figure 6).

The subcellular localization of lncRNAs is closely related to their functional mode and regulatory mechanism.^37^ It has been proven that lncRNAs located in the nucleus can transcriptionally regulate their downstream target genes through interactions with specific proteins.^57^ To further investigate the mechanism by which *AC018926.2* influences the expression of *ITGA2*, we used bioinformatics analysis to predict potential proteins that might interact with *AC018926.2* and potential transcription factors of *ITGA2*, and unfortunately there was no intersection of the both above mentioned. Among the predicted proteins that might interact with *AC018926.2*, DNMT3B, DNMT1, PARP1 and SUZ12 have been reported to be associated with the transcription of *ITGA2*.^46^, ^47^, ^48^ Therefore, they were selected for subsequent experiments, and qRT‐PCR assays demonstrated that the knockdown of DNMT3B, PARP1 and SUZ12 significantly affected the level of *ITGA2* in PDLSCs (Figure 7C). RIP assays confirmed the specific binding of *AC018926.2* to PARP1 but not to DNMT3B or SUZ12 (Figure 7D). The rescue experiment verified that *AC018926.2* transcriptionally promoted *ITGA2* expression in PDLSCs by recruiting PARP1 (Figure 7F). Nevertheless, this regulation may not be direct, and more mechanistic investigations are required to further confirm this hypothesis. PARP1 has been reported to regulate gene expression by interacting with chromatin, chromatin modifiers, or transcription factors.^58^ Meanwhile, recent studies have indicated that PARP1 is also an RNA‐binding protein. Man et al.^59^ reported that PARP1 was directly recruited to the promotor of *KLF2* by lncRNA *STEEL*, resulting in the upregulation of *KLF2* mRNA expression. Fang et al.^60^ illustrated that lncRNA *MZF1‐AS1* bound to PARP1 to enhance its interaction with E2F1, leading to the activation of E2F1 and positively affecting downstream target genes. Interestingly, E2F1 is one of the transcription factors of *ITGA2* predicted by AnimalTFDB (http://bioinfo.life.hust.edu.cn/AnimalTFDB4/#/). PARP1 is located in the nucleus, which is consistent with the nuclear localization of *AC018926.2*. We assumed that *AC018926.2* that is enriched in the nucleus might independently recruit PARP1 to target promoters or facilitate its interaction with the transcription factor of *ITGA2*. The specific mechanisms will be further explored in our future work.

It should be noted that this is a relatively preliminary exploration on the effect of a high‐fat condition on the osteogenic differentiation potential of PDLSCs. Although we identified for the first time that *AC018926.2* served as an active regulator in the process of PA exposure‐impaired osteogenic differentiation of PDLSCs, and *AC018926.2* was positively correlated with the activity of ITGA2/FAK/PI3K/AKT pathway, it is not clear whether *AC018920.2* changed in abundance due to altered transcriptional regulation of *AC018920.2* or simply due to differing content of cell types following 14 days of treatment with PA at present. Nevertheless, our results clarified that the forced expression of *AC018926.2* was able to reverse PA‐compromised PDLSC osteogenic differentiation, thus indicating that *AC018926.2* is an important target for improving osteogenic differentiation potential of cells exposed to a PA condition. To go deeply into the research, we will try to use methods such as single‐cell sequencing to further explore whether the reduced expression of *AC018926.2* is due to the transcriptional downregulation in all cells or due to the altering abundance of individual cell types present in these cultures.

**FIGURE 8:**
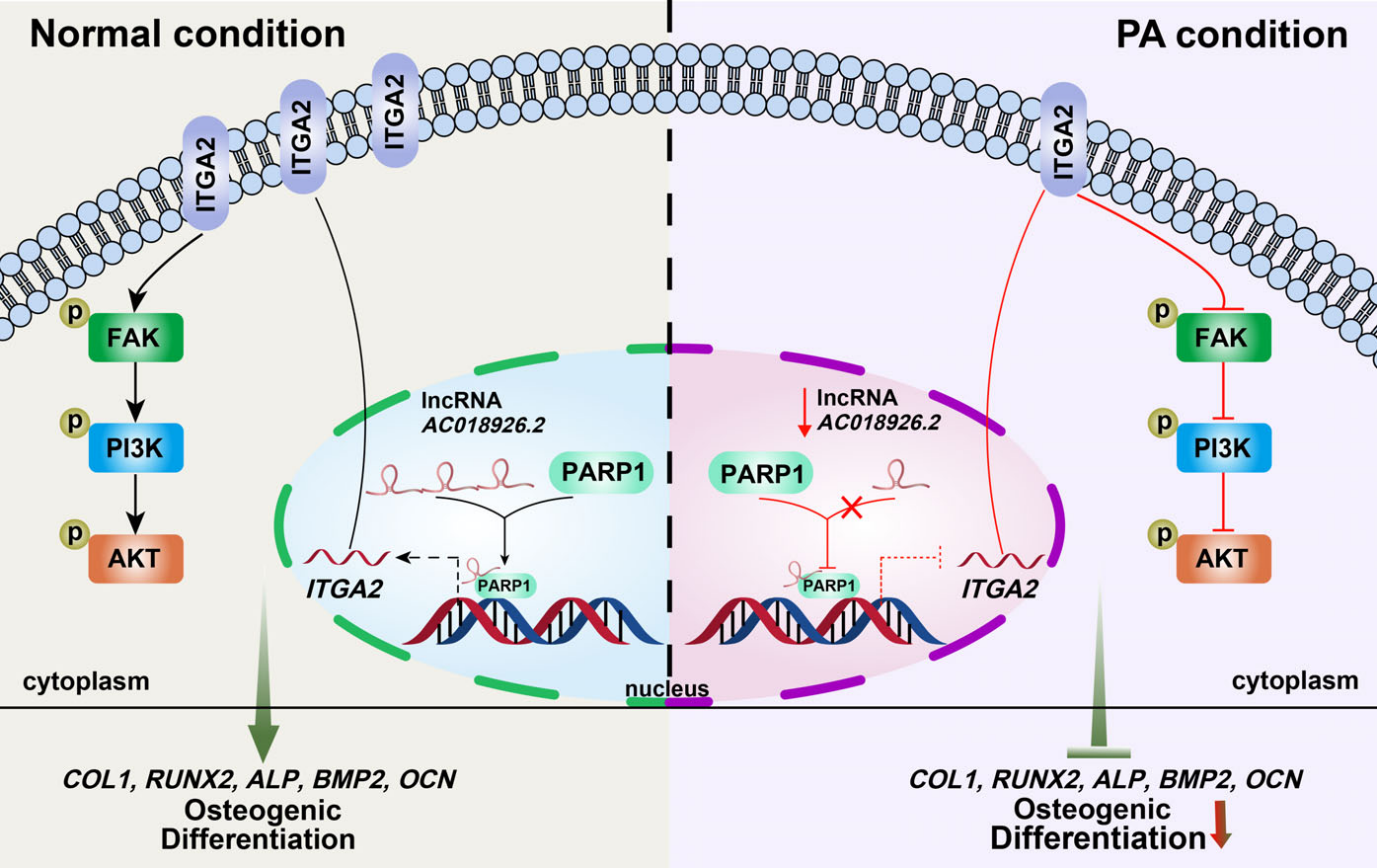
A proposed model illustrating the function and mechanism of *AC018926.2* in PDLSC osteogenic differentiation with PA exposure.

In conclusion, our study identified and validated that the lncRNA *AC018926.2* plays a crucial role in the PA exposure‐compromised osteogenic differentiation of PDLSCs for the first time (Figure 8). The overexpression of *AC018926.2* activated the ITGA2/FAK/PI3K/AKT pathway by transcriptionally upregulating *ITGA2* through interacting with PARP1 and then reversed the damaged osteogenic potential of PDLSCs caused by PA exposure. Although the underlying mechanism by which *AC018926.2* regulates *ITGA2* still needs to be further explored, the present study indicates a novel therapeutic target for periodontium recovery in obese individuals.

## AUTHOR CONTRIBUTIONS

Hong‐Lei Qu and Li‐Juan Sun: Data collection and analysis, Manuscript writing. Li Xuan, Fen Liu and Hai‐Hua Sun: Statistical analysis and Manuscript writing. Xiao‐Tao He, Dian Gan, Yuan Yin and Bei‐Min Tian: Collection of data and Technical assistance. Fa‐Ming Chen and Rui‐Xin Wu: Design of study, Supervision, Financial support and Writing revision. All authors read and approved the final manuscript.

## FUNDING INFORMATION

This work was supported by grants from the Major Research Program of the National Natural Science Foundation of China (Beijing, Subproject No. 82130026) and the National Natural Science Foundation of China (Beijing, Nos. 82170958, 82101013, 82201061 and 82170926).

## CONFLICT OF INTEREST STATEMENT

The authors declare no potential conflicts of interest to declare.

## Supporting information


**Data S1:** Supporting Information

## Data Availability

The lncRNA microarray data are available in the GEO databases (accession number GSE218434).
