# Supplementary material for: Long non‐coding RNA AC018926 .2 regulates palmitic acid exposure‐compromised osteogenic potential of periodontal ligament stem cells via the ITGA2/FAK/AKT pathway
Source: Cell Prolif. 2023 Jan 31;56(8):e13411. doi: 10.1111/cpr.13411 (PMC10392068; doi:10.1111/cpr.13411)
Supplement: Supplementary file 1 — Data S1: Supporting Information [file CPR-56-e13411-s001.docx]

**Supplementary information**

**Supplementary Materials and Methods**

**Isolation and culture of PDLSCs**

Fresh teeth extracted for orthodontic reasons were obtained from 6 healthy donors between 18 and 30 years old who were free of systemic diseases, hereditary diseases, and acute infections. PDLSCs were isolated as previously described.^1^ This study was approved by the Ethics Committee of the School of Stomatology, Fourth Military Medical University, and all donors were fully informed and signed informed consent. Briefly, the teeth were repeatedly rinsed using sterile phosphate-buffered saline (PBS; Corning, New York, USA), and gingival tissue was removed. PDL tissues were gently scraped from the middle 1/3 of the root surface and digested with 0.3% type I collagenase (Sigma-Aldrich, St. Louis, USA) for 45 min. The tissue pieces were then maintained in minimum essential medium alpha (α-MEM; Gibco, New York, USA) containing 10% fetal bovine serum (FBS; Sijiqing, Hangzhou, China) and 1% penicillin-streptomycin (Invitrogen, Carlsbad, CA, USA) in an incubator with a humidified atmosphere of 5% CO_2_ at 37°C. The medium was exchanged every 2 days. The primary cells were digested for passage when they reached a confluence of 80%–90%. Cells between passages 3 and 5 were used for *in vitro* experiments.

**Flow cytometry analysis**

The immunophenotypes of PDLSCs were assessed using flow cytometry analysis.^1^ Briefly, PDLSCs were transferred and resuspended into 1.5-mL Eppendorf tubes (Invitrogen) at a density of 5 × 10^5^ cells per tube. Then, the cells in each tube were incubated with phycoerythrin (PE)-or fluorescein isothiocyanate (FITC)-conjugated monoclonal antibodies against human CD34, CD44, CD45, CD90, CD105 and CD146 (all from eBioscience, San Diego, USA) in the dark for 1 hour. Cells without incubated antibodies were used as blank controls. After washing and resuspending with PBS, the immunophenotypes of these cells were identified utilizing a Beckman Coulter Epics AL cytometer (Beckman Counter, Fullerton, USA).

**Colony‑forming unit (CFU) assay**

To detect the capacity of PDLSCs to form colonies, 1 × 10^3^ PDLSCs were seeded in a 100-mm-diameter culture dish and cultured in complete α-MEM medium for 14 days. The medium was exchanged every 2 days. Then, the cells were fixed with 4% paraformaldehyde (Invitrogen) for 30 min at room temperature and stained with 0.5% crystal violet solution (Sigma-Aldrich) for 10 min. Aggregates comprising at least 50 cells were considered as colonies. Images of cell colonies were taken using an inverted microscope (Olympus, Tokyo, Japan).

**Cell Counting Kit‑8 (CCK‑8) assay**

A CCK‑8 assay was performed to measure the reproductive capacity of PDLSCs with a Cell Counting Kit (Invitrogen). The cells were seeded into 96-well culture plates (Invitrogen) at a density of 1 × 10^3^ cells per well. After 24 hours of cell adherence, the complete medium was replaced by 200 μL of new medium containing 20 μL of CCK-8 reagent. The cells were placed in the incubator for 2 hours at 37 °C, and then the absorbance at 450 nm was assessed with a microplate reader (Infinite M200 Pro, Tecan, Switzerland). During the 8-day period of culture, all steps were conducted at stipulated time points each day.

**Adipogenic differentiation assay**

For adipogenic induction, PDLSCs were cultured in adipogenic medium (a-MEM containing 10% FBS, 0.5 mM 3-isobutyl-1-methylxanthine, 5 μg/ml insulin, 1 μM dexamethasone and 200 mM indomethacin (Cyagen Biosciences, Guangzhou, China)) for 21 days. The medium was exchanged every 2 days. After adipogenic induction, Oil Red O (Sigma-Aldrich) staining was conducted in order to detect lipid droplets. The stained lipid droplets were then observed and photographed using an inverted microscope (Olympus).

**Chondrogenic differentiation assay**

For chondrogenic induction, 3-4 × 10^5^ cells were transferred into 15-mL centrifuge tubes (Invitrogen) and resuspended in 0.5 mL chondrogenic medium (Cyagen). Then, the cells were kept in an incubator without movement until cell spheres were visible at the bottom of the tube (normally 24-48 hours). The medium was exchanged every 2 days. Continuous induction was performed until the diameter of the cartilage spheres reached 1.5-2 mm, and the samples were then sectioned and stained with Alcian blue.

**Cell osteogenic induction and PA exposure**

For osteogenic differentiation, PDLSCs were cultured in osteogenic medium (a-MEM containing 10% FBS, 0.1 mmol/L dexamethasone, 10 mmol/L b-sodium glycerophosphate, and 50 mg/mL ascorbic acid (Cyagen)) for 14 or 21 days. During this process, in order to build a high-fat condition, PA and its solvent control (FA-free bovine serum albumin, BSA) that were purchased from Kunchuang Biotechnology (Xi'an, China) were used. PA was added to the osteogenic medium (PA group) to adjust the final concentration of PA to 200 μM, and the control group was treated with the same amount of BSA. The medium was exchanged every 2 days.

**Alkaline phosphatase (ALP) staining and quantification**

After 14 days of osteogenic induction, the cells were fixed with 4% paraformaldehyde for 30 min at room temperature, followed by ALP staining using a BCIP/NBT ALP Color Development Kit (Beyotime, Haimen, China) for 30 min in the dark. Images were captured under a microscope (Olympus) after rinsing. Meanwhile, the medium supernatant from the samples was collected for ALP quantification using an ALP activity detection kit (Nanjing Jiancheng Bioengineering Institute, Nanjing, China).

**Alizarin red S staining and quantification**

After osteogenic induction for 21 days, the cells were fixed for Alizarin red S staining using 2% Alizarin red solution (Cyagen). The samples were incubated in the solution for 5-10 min in the dark, and the matrix mineralization was visualized and photographed under a microscope (Olympus). To measure the level of mineralization secreted from PDLSCs, the calcium nodules were dissolved in 2% cetylpyridine (Sigma-Aldrich) and the absorbance was observed at 560 nm.

**Quantitative real-time polymerase chain reaction (qRT-PCR)**

Total RNA was extracted from PDLSCs using TRIzol reagent (Invitrogen), and 1 μg of the RNA was reverse transcribed into cDNA with the PrimeScript RT Reagent Kit (TAKARA, Tokyo, Japan). The cDNA templates were measured by qRT-PCR assays using specific primers and a SYBR Green Kit (TAKARA) on the CFX96 Touch Real-Time PCR Detection System (Bio-Rad, Hercules, CA, USA). All primers were synthesized by TSINGKE (Beijing, China), and the detailed sequences used are provided in Table S1. Either *β-actin*, *GAPDH* or *U6* served as the internal reference, and the relative expression of each gene was calculated using the 2^-ΔΔ (ct)^ method.

**Western blot analysis**

The cells were collected and lysed by RIPA buffer (Beyotime) supplemented with proteinase and phosphatase inhibitor cocktail (Beyotime). Protein concentrations were determined by a bicinchoninic acid (BCA) assay kit (Biotime). Protein samples of equal quality (approximately 20 μg) were separated by 10% SDS-PAGE gels (Biotime), and transferred to 0.45 μm PVDF membranes (Millipore, Billerica, MA, USA). The membranes were blocked with 5% skim milk (Biotime) at room temperature for 2 hours and then incubated with primary antibodies at 4 ℃ overnight. After 2 hours of exposure to corresponding horseradish peroxidase (HRP)-conjugated secondary antibodies, the blots were visualized using enhanced chemiluminescent ECL reagent (Millipore). The gray value of target proteins was measured with ImageJ software, and β-actin was used as the internal control for normalization. The primary antibodies used for Western blotting were as follows: antibodies targeting osteogenesis-related proteins COL1 (dilution 1:1000, Proteintech; 14695-1-AP), RUNX2 (1:1000, Cell Signaling Technology; #12556), ALP (1:1000, Abcam; ab65834) and BMP2 (1:1000, Proteintech; 66383-1-IG); antibodies targeting ITGA2/FAK/PI3K/AKT pathway-related proteins ITGA2 (1:1000, Abcam; ab133557), FAK (1:1000, Cell Signaling Technology; #13009), phospho-FAK (Tyr397; 1:1000, Cell Signaling Technology; #8556), AKT (1:1000, Cell Signaling Technology; #9272) and phospho-AKT (Ser473; 1:1000, Cell Signaling Technology; #9271); and a specific antibody against β-actin (1:1000, Cell Signaling Technology; #8457).

**Lentivirus infection**

Recombinant lentiviruses containing the whole sequence of *AC018926.2* and the scrambled control were synthesized by HANBIO (Shanghai, China). When the PDLSCs reached 40-50% confluence, they were transfected with lentivirus that overexpressed *AC018926.2* at a MOI of 50 in combination with 5 μg/ml polybrene for 24 hours, and then the medium was replaced with regular complete medium. Seventy-two hours after transfection, puromycin of 2 μg/ml was added to the culture medium for 1 week to select transfected cells. qRT-PCR assays were conducted to confirm the efficiency of *AC018926.2* overexpression.

**RNA interference**

Small interfering RNAs (siRNAs) targeting *AC018926.2*, ITGA2, FAK, DNMT1, DNMT3B, SUZ12, PARP1 and the negative control (si-NC) were provided by GenePharma (Shanghai, China). The sequences are listed in Table S2. Transfection of PDLSCs was conducted using Lipofectamine 2000 (Invitrogen) and Opti-MEM (Gibco) according to the manufacturer’s instructions. The final concentration of siRNAs was 100 nM. Cells were lysed at 48 and 72 hours after transfection for RNA and protein analyses, respectively, to validate the knockdown efficiency. During osteogenic induction and PA administration, siRNA transfection was performed every 5 days to maintain the interference efficiency until the cells were treated for a total of 14 or 21 days.

**LncRNA microarray analysis**

Three groups of PA-exposed PDLSCs (PA group) and paired BSA-exposed PDLSCs (Ctrl group) were collected and lysed, and total RNA was extracted using TRIzol Reagent (Invitrogen). The samples were then amplified and labeled with fluorescent cRNAs. After purifying the labeled cRNAs, they were subjected to a lncRNA microarray using an Arraystar Human lncRNA Array (ArrayStar, Rockville, MD, USA). The microarray experiment and analysis were conducted by Kangcheng Biotech Inc. (Shanghai, China). The microarray data can be accessed by the accession number GSE218434 in the GEO databases. lncRNAs with a fold change > 1.5 and p value < 0.05 were considered differentially expressed. A volcano plot was created to show the differences in gene expression between the two groups. A heatmap was generated to indicate the top 10 downregulated and top 10 upregulated lncRNAs in the PA group compared to those in the Ctrl group.

**Subcellular fractionation**

Nuclear and cytoplasmic fractions of PDLSCs were separated using the Nuclear/Cytoplasmic Isolation kit (Thermo Fisher Scientific, Carlsbad, CA, USA) according to the manufacturer’s protocol. RNA was extracted from nuclear and cytoplasmic fractions and then reverse transcribed into cDNA. Subsequently, qRT-PCR was performed in order to detect the *AC018926.2* expression level in the two fractions, with *GAPDH* serving as an internal reference for the cytoplasm and *U6* as an internal reference for the nucleus.

**RNA sequencing and bioinformatics analysis**

Total RNA isolated from *AC018926.2* knockdown PDLSCs (si-*AC018926.2* group) and their corresponding control cells (si-NC group) was subjected to commercial RNA sequencing (Gene Denovo Biotechnology Co, Guangzhou, China). Briefly, total RNA was extracted using a TRIzol reagent kit (Invitrogen), and eukaryotic mRNA was enriched by Oligo (dT) beads. Then, the enriched mRNA was fragmented and reverse transcribed into cDNA. After purification, the cDNA fragments were end repaired, poly(A) added, and ligated to Illumina sequencing adapters. The ligation products were then size selected, amplified, and sequenced using Illumina Novaseq 6000. Differentially expressed genes were identified with thresholds of fold change > 2 and p < 0.05. A heatmap was generated to show the differences in gene expression between the two groups. Pathway enrichment analysis was conducted using KEGG to show the pathways possibly affected by *AC018926.2* knockdown.

**RNA immunoprecipitation (RIP)**

RIP experiments were performed using an RNA Immunoprecipitation Kit (BersinBio, Guangzhou, China) according to the manufacturer’s protocol. In brief, 1×10^7^ PDLSCs were lysed with RIP lysis buffer, and the cell extracts were incubated with magnetic beads conjugated to specific antibodies at 5 μg per reaction at 4°C overnight. The immunoprecipitated RNAs were isolated and then reverse transcribed into cDNA. RT-qPCR was used to measure the expression level of *AC018926.2* in each group. The antibodies for the RIP assays were as follows: antibody targeting DMNT3B (Cell Signaling Technology; # 57868), antibody targeting PARP1 (Proteintech; 13371-1-AP), antibody targeting SUZ12 (Proteintech; 20366-1-AP) and control antibody targeting IgG (BersinBio).

**Statistical analysis**

Statistical analysis was performed using GraphPad Prism 8 software. All data are presented as the mean ± standard deviation (SD). Each experiment was repeated at least 3 times. Student’s t test was employed for comparisons between two groups, and for multiple comparisons, a one-way analysis of variance (ANOVA) followed by Tukey’s test was conducted. P < 0.05 was considered statistically significant.

**Reference**

1. Li X, He XT, Kong DQ, et al. M2 macrophages enhance the cementoblastic differentiation of periodontal ligament stem cells via the Akt and JNK pathways. *Stem Cells.* 2019;37:1567-1580.

**Supplementary Tables**

**Table S1.** **Sequences of gene-specifc primers for qRT-PCR in the present study**

| Genes | Forward sequence (5’-3’) | Reverse sequence (5’-3’) |
| --- | --- | --- |
| *ALP* | AACATCAGGGACATTGACGTG | GTATCTCGGTTTGAAGCTCTTCC |
| *COL1* | GAGGGCCAAGACGAAGACATC | CAGATCACGTCATCGCACAAC |
| *OCN* | CCCAGGCGCTACCTGTATCAA | GGTCAGCCAACTCGTCACAGTC |
| *RUNX2* | TGGTTACTGTCATGGCGGGTA | TCTCAGATCGTTGAACCTTGCTA |
| *BMP2* | ACTACCAGAAACGAGTGGGAA | GCATCTGTTCTCGGAAAACCT |
| *β-actin* | CTCCATCCTGGCCTCGCTGT | GCTGTCACCTTCACCGTTCC |
| *GAPDH* | GGGAAACTGTGGCGTGAT | GAGTGGGTGTCGCTGTTGA |
| *U6* | GGAACGATACAGAGAAGATTAGC | TGGAACGCTTCACGAATTTGCG |
| *BIG-lncRNA-582* | GCGGGTTCTTCAGGAGGTATCTA | GTCCTCGGGGCAATCACAC |
| *AC018926.2* | ATCACCAGTTCAGCAAAGTCAG | AGAATCCATCCAGAAGGAAAGA |
| *FGF7* | GTTTAGGGCACATCGCAGGTT | ACTTTCCACCCCTTTGATTGC |
| *SOX9-AS1* | AAATGCCATGACTCCCACTAT | TTCATACAACCACAGGACCAC |
| *INTS6-AS1* | ACATAGTCCAGGGTCACAAAGC | GTCACCGTCTTCTATCTCCACAT |
| *MIR100HG* | GCGTTTCTTGTCTTCTATTGTGC | GAAGAACAATGGATTTGGGATG |
| *AC008734.2* | CCAAGGCAGAACATAGTGATT | GCAAAAGAACTGAAAGACCG |
| *G090757* | AGAACCCTCAACAATACACCAGA | CTCAAAGTTTCTTCTTGCCATCT |
| *AL121845.1* | AAACACTTGGGGAGCCCTGAA | CACTCACCATGAGGACATTCCATC |
| *AL512444.1* | TGGACAGAAGAAATGAACAAGAGG | CACATCCATCCCAGAAACATCTT |
| *ITGA2* | ACAAGTGGGATTCAGTGCAGATT | TGCAGCCACAGAGTAACCTAAAT |
| *PARP1* | CGGAGTCTTCGGATAAGCTCT | TTTCCATCAAACATGGGCGAC |
| *DNMT1* | GTGGGGGACTGTGTCTCTGT | TGAAAGCTGCATGTCCTCAC |
| *DNMT3B* | TTGAATATGAAGCCCCCAAG | GGTTCCAACAGCAATGGACT |
| *SUZ12* | AGGCTGACCACGAGCTTTTC | GGTGCTATGAGATTCCGAGTTC |

**Table S2. Sequences of siRNAs in this study**

| Genes | Sense Strand (5’-3’) | Antisense Strand (5’-3’) |
| --- | --- | --- |
| si-*AC018926.2* | GUUGGUUGAUCAGUCAGAUTT | AUCUGACUGAUCAACCAACTT |
| si-FAK | CAGGUGAAGAGCGAUUAUATT | UAUAAUCGCUCUUCACCUGTT |
| si-ITGA2 | CGGCCAGAUAGUGCUAUAUTT | AUAUAGCACUAUCUGGCCGTT |
| si-DNMT1 | GUCCCAAUAUGGCCAUGAAdTdT | UUCAUGGCCAUAUUGGGACdTdT |
| si-DNMT3B | AGAUGACGGAUGCCUAGAGdTdT | CUCUAGGCAUCCGUCAUCUdTdT |
| si-PARP1 | CGACCUGAUCUGGAACAUCAA | UUGAUGUUCCAGAUCAGGUCG |
| si-SUZ12 | CAUCGAAACUCCAGAACAATT | UUGUUCUGGAGUUUCGAUGTT |
| si-NC | UUCUCCGAACGUGUCACGUTT | ACGUGACACGUUCGGAGAATT |

**Supplementary Figures**

**
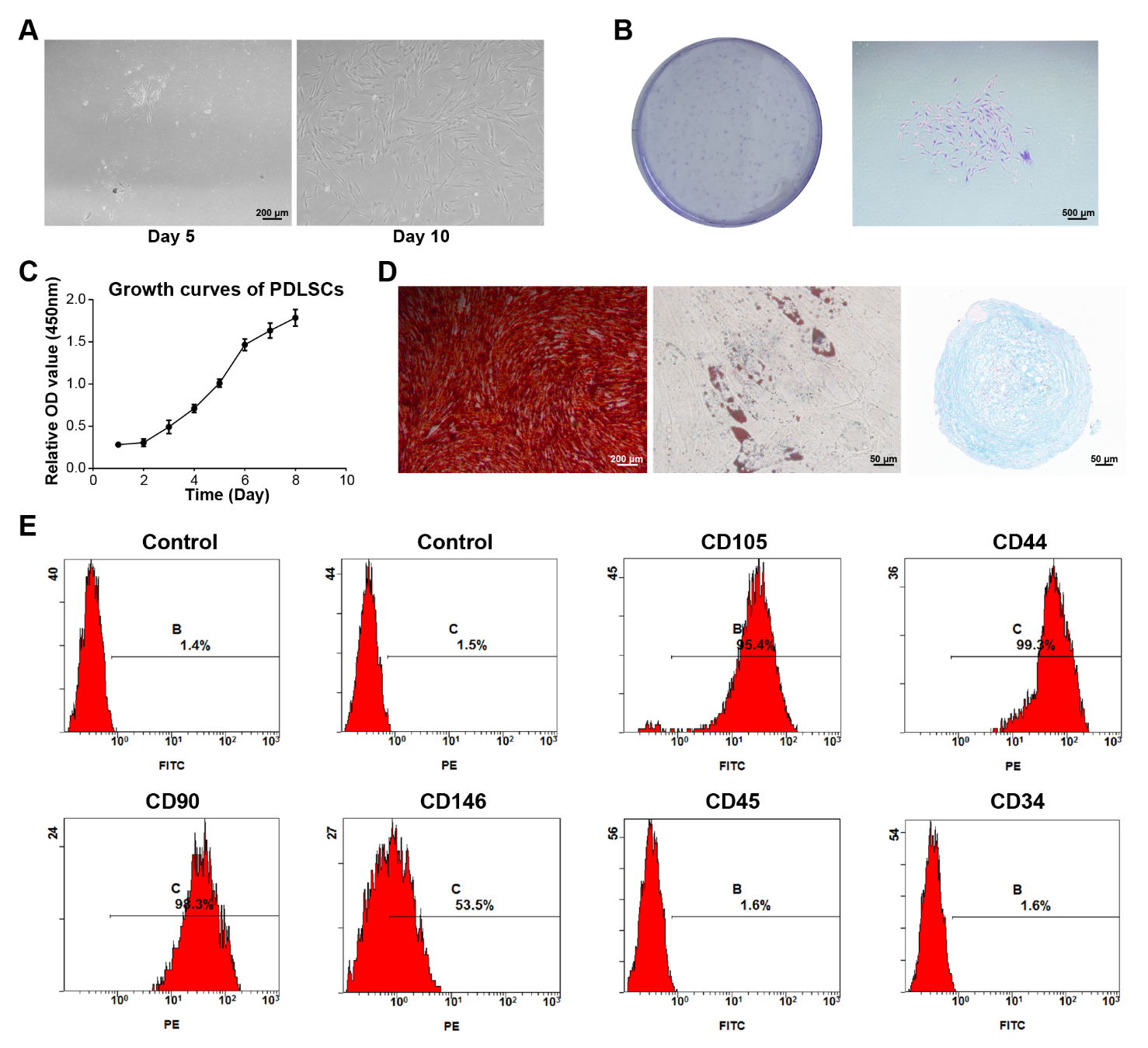
**

**Figure S1.** Isolation and characterization of PDLSCs. (A) Primary PDLSCs were obtained from PDL tissues observed on day 5 and day 10 (scale bar: 200 μm). (B) Cell colonies formed by PDLSCs were observed in a general view and in a macroscopic view (scale bar: 500 μm). (C) Proliferative activity of PDLSCs detected by CCK-8 assay during culture for 8 days. (D) Alizarin red S staining (left; scale bar = 200 µm), Oil red O staining (middle; scale bar = 50 µm) and Alcian blue staining (right; scale bar = 50 µm) of PDLSCs following osteogenic, adipogenic or chondrogenic induction. (E) Surface markers of PDLSCs assessed by flow cytometry analysis.

**
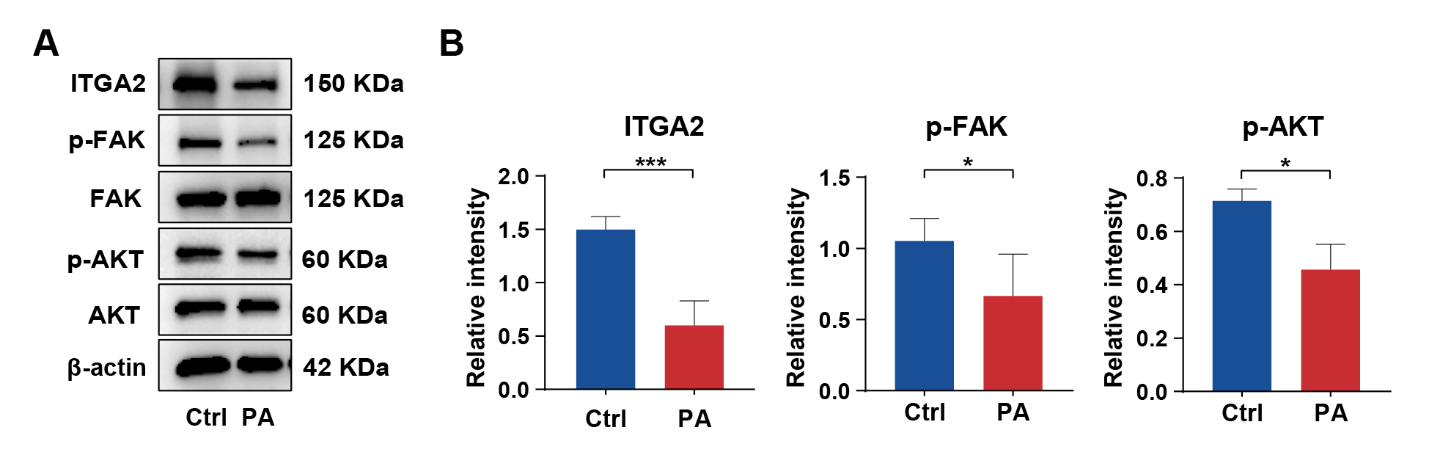
**

**Figure S2.** PA exposure inhibited the activity of the ITGA2/FAK/PI3K/AKT pathway. The cells were incubated in osteogenic medium with BSA (Ctrl) or osteogenic medium with PA for 14 days.

(A, B) The effect of PA exposure on protein expression levels of ITGA2, p-FAK, total FAK, p-AKT, and total AKT in PDLSCs determined by Western blot analysis. The relative intensity of ITGA2 was normalized to β-actin, and the relative intensity of p-FAK and p-AKT was normalized to respective total FAK and total AKT. All experiments were performed with 3 biological replicates. Data are presented as the mean ± SD (n = 3). **p* < 0.05 and ****p* < 0.001 represent significant differences between the indicated columns.

**
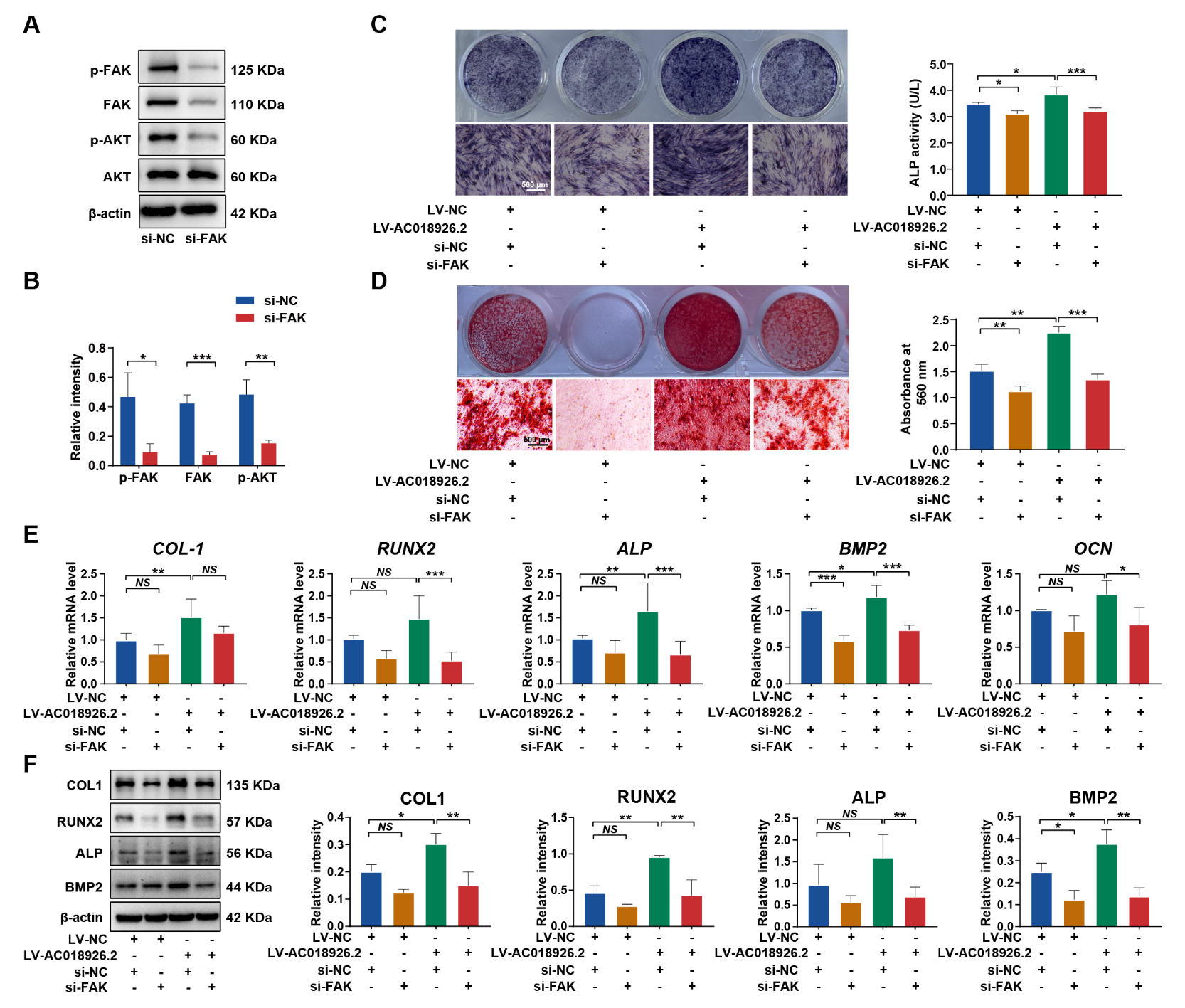
**

**Figure S3.** Inactivation of the FAK/PI3K/AKT pathway reversed the *AC018926.2* overexpression-mediated enhancement of PDLSC osteogenic differentiation with PA exposure. (A, B) Western blot analysis was conducted to detect p-FAK, total FAK, p-AKT, and total AKT protein levels in the si-NC and si-FAK groups. The relative intensity of p-FAK and FAK was normalized to β-actin, and the relative intensity of p-AKT was normalized to total AKT. The cells were incubated in normal medium transfected with si-FAK. (C) Representative images of ALP staining and quantification of ALP activity in PDLSCs after osteogenic induction for 14 days (scale bar: 500 µm). (D) Representative images of Alizarin red S staining and quantitative analysis of the calcium mineral deposits formed by PDLSCs after osteogenic induction for 21 days (scale bar: 500 µm). (E) The effect of FAK inhibition on the expression levels of the osteogenesis‑related genes *COL1*, *RUNX2*, *ALP*, *BMP2* and *OCN* in *AC018926.2* overexpressed PDLSCs following 14 days of osteogenic induction measured by qRT-PCR. (F) The effect of FAK inhibition on the osteogenesis-related proteins COL1, RUNX2, ALP and BMP2 in *AC018926.2* overexpressed PDLSCs following 14 days of osteogenic induction determined by Western blot analysis. The cells were incubated in osteogenic medium with PA and co-transfected with LV-*AC018926.2* and si-FAK (C-F). All experiments were performed with 3 biological replicates. Data are presented as the mean ± SD (n = 3). **p* < 0.05, ***p* < 0.01, and ****p* < 0.001 represent significant differences between the indicated columns, while *NS* represents no significant difference.
